# Supplementary material for: Nanoporous Cauliflower-like Pd-Loaded Functionalized Carbon Nanotubes as an Enzyme-Free Electrocatalyst for Glucose Sensing at Neutral pH: Mechanism Study
Source: Sensors (Basel). 2022 Apr 1;22(7):2706. doi: 10.3390/s22072706 (PMC9002983; doi:10.3390/s22072706)
Supplement: Supplementary file 1 [file sensors-22-02706-s001.zip › sensors-1658745-supplementary.pdf]

Supplementary Materials

Nanoporous Cauliflower-like Pd-Loaded Functionalized Carbon Nanotubes as an Enzyme-free Electrocatalyst for Glucose Sensing at Neutral pH: Mechanism Study

Abdelghani Ghanam [1,2], Naoufel Haddour [2], Hasna Mohammadi [1], Aziz Amine [1,*], Andrei Sabac [2] and François Buret [2]

[1] Chemical Analysis and Biosensors Group, Laboratory of Process Engineering and Environment, Faculty of Science and Techniques, Hassan II University of Casablanca, B.P 146, Mohammedia, Morocco; abdelghani.ghanam@ec-lyon.fr (A.G.)

[2] Laboratoire Ampère, Ecole Centrale de Lyon, Université de Lyon, CNRS, UMR 5005, 69130 Ecully, France; naoufel.haddour@ec-lyon.fr (N.H.)

* Correspondence: a.amine@univh2m.ac.ma; Tel.: +212-661-455198; Fax.: +212-523-315353

Keywords: cauliflower-like Pd, enzyme-free electrocatalyst, glucose, mechanism, electrodeposition, functionalized carbon nanotube

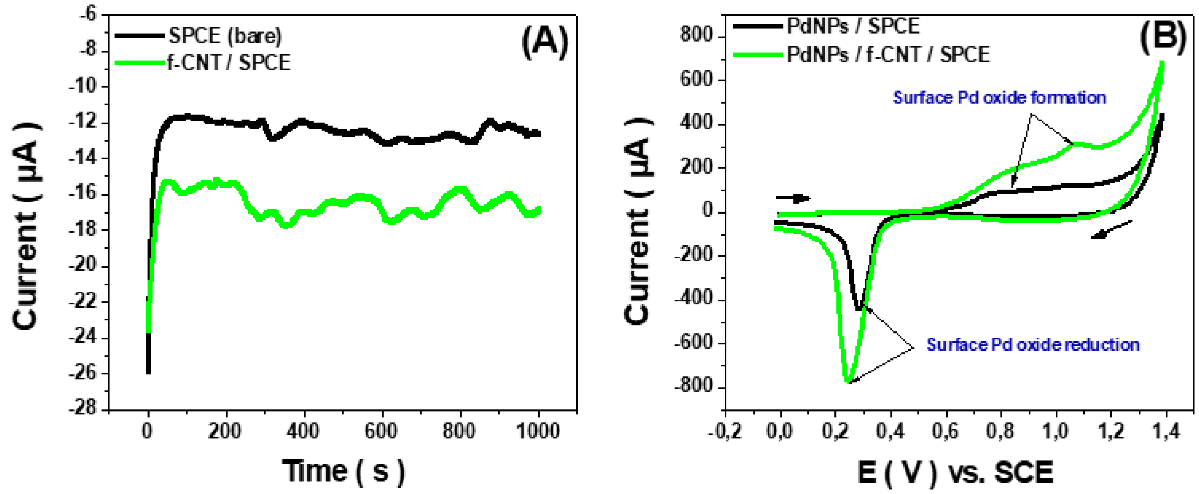

Figure S1. (**A**) electrodeposition chronoamperograms of PdNS under -0.2 V vs. SCE for 1000 s in a 0.05 M $HClO_4$ and 0.25 M H_2SO_4 solution containing 1 mM Pd^{2+} over bare SPCE (**black line**) and f-CNT/SPCE (**green line**). (**B**) First scan, of five successive scans, related to the polarization of PdNS modified SCPE and f-CNTs/SPCE recorded by CVs in 0.5 M H_2SO_4 solution, scan rate 100 mV s^{-1}.

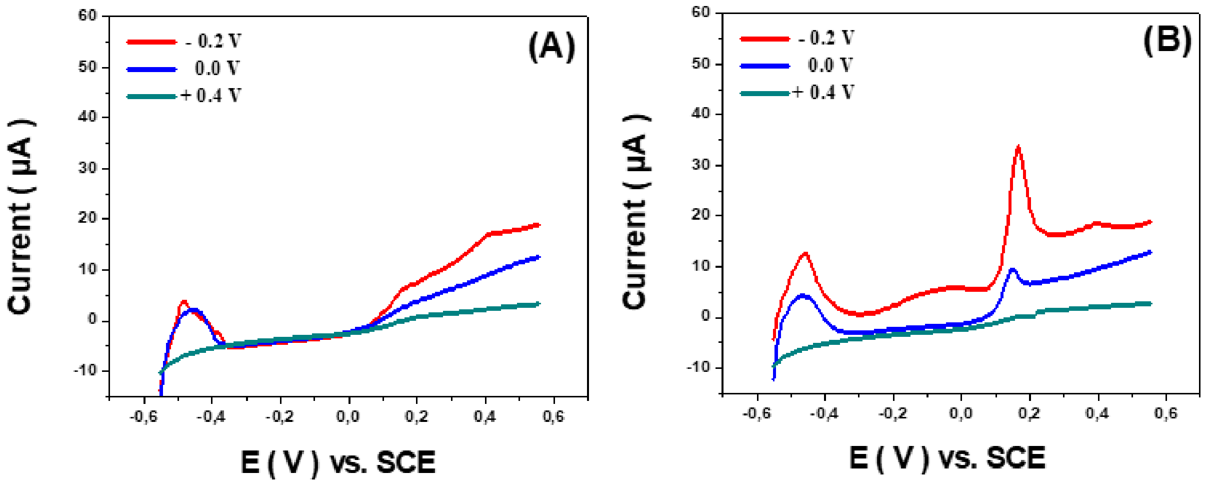

Figure S2. LSVs at PdNS/f-CNT/SPCE recorded after Pd electrodeposition at −0.2, 0.0, and +0.4 V vs. SCE in 0.1 M PBS (pH 7.4) without (**A**) and in the presence of 20 mM of glucose (**B**). Scan rate 10 mV s^{-1}

1.1. Effect of Electrodeposition Potential

This approach uses the value of 0.424 mC cm^{-2} as the charge density associated with the reduction of one monolayer of PdO. However, knowing the potential range corresponding accurately to the formation of one monolayer of PdO in a cyclic voltammetry transient is highly recommended for applying this methodology [1,2]. Indeed, the real surface area of the PdNS could be calculated by evaluating the charge consumed during PdO electroreduction in the cathodic scan [2,3]. Therefore, the real surface area (RSA) can be estimated using the following equation:

$$\mathrm{RSA} = \frac{Q^{O}{}_{red}}{\theta_O \, Q^{O}{}_{red,ML,S}}$$

where Q^{O}_{red} represents the charge required for oxygen adsorption, θ_O is the surface coverage with surface oxide (adsorbed oxygen) and $Q^{O}_{red,ML,S}$ is the charge due to the reduction of a monolayer (ML) of surface oxide per unit area (0.424 mC cm^{-2}) . As previously reported, the values of Q^{O}_{red} and θ_O depend on the potential of electrode polarization and the time scale of the experiment, i.e. scan rate (mV s^{-1}) in voltammetric measurements [3].

Indeed, cyclic voltammetry profiles were performed at a scan rate of 100 mV s^{-1} to illustrate PdO formation and reduction. Thereafter, Q^{O}_{red} was calculated on the basis of the integral discharge area of PdO formation during the cathodic scan on these CV profiles.

1.2. Mechanism Proposed for Glucose Oxidation on PdNS Catalyst in Neutral pH

Figure S3 shows the E-pH equilibrium diagram for the system Pd-water at 25°C, adapted from M. Pourbaix, Atlas of Electrochemical Equilibria in Aqueous Solution [4], and their corresponding theoretical reactions.

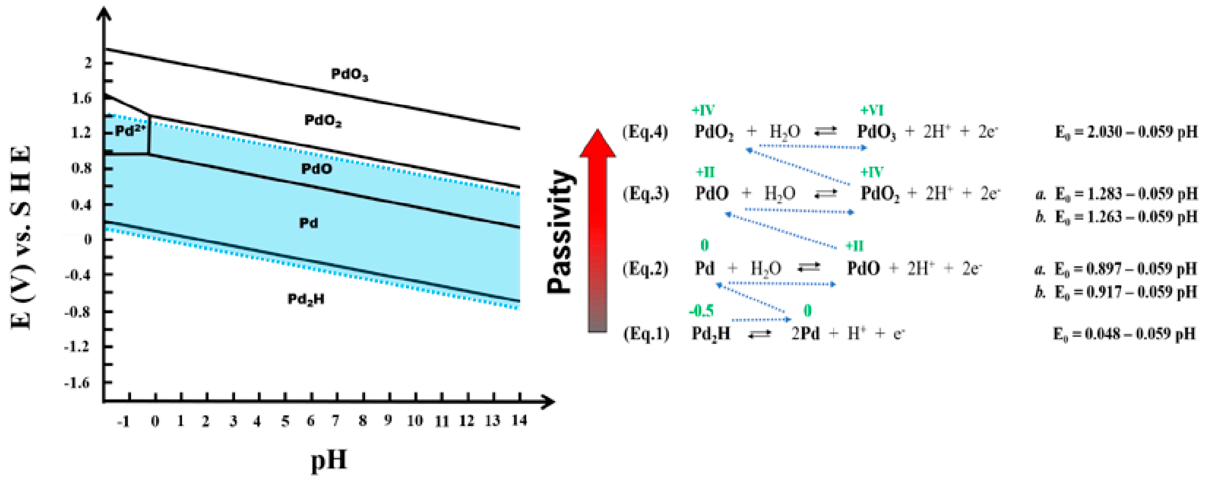

Figure S3. E-pH equilibrium diagram for the system Pd-water, at 25 °C, adapted from M. Pourbaix, Atlas of Electrochemical Equilibria in Aqueous Solution [4], and their corresponding theoretical reactions and equilibrium formulas.

The potentials in the Pourbaix diagram are expressed with respect to a standard hydrogen electrode (SHE). However, the values found are converted to be versus a saturated calomel electrode (SCE), saturated with 3M KCl, and presented as follows:

**E (PdO_3/PdO_2)** = 2.030 – 0.0591 **pH** = 1.593 vs. SHE = + 1.353 vs. SCE	(1)
**E (PdO_2/PdO)^a** = 1.283 – 0.0591 **pH** = 0.846 vs. SHE = + 0.606 vs. SCE	(2)
**E (PdO_2/PdO)^b** = 1.263 – 0.0591 **pH** = 0.826 vs. SHE = + 0.586 vs. SCE	
**E (PdO/Pd)^a** = 0.897 – 0.0591 **pH** = 0.46 vs. SHE = + 0.22 vs. SCE	(3)
**E (PdO/Pd)^b** = 0.917 – 0.0591 **pH** = 0.48 vs. SHE = + 0.24 vs. SCE	
**E (Pd/Pd_2H)** = 0.048 – 0.0591 **pH** = - 0.389 vs. SHE = - 0.629 vs. SCE	(4)

In Equation (2), involving PdO, the letter **(a)** refers to hydrated PdO or palladous hydroxide $Pd(OH)_2$, whereas the letter **(b)** refers to anhydrous PdO [4]. Besides, the Pd(II) oxides are reported to be either anhydrous PdO or hydrous, i.e., $Pd(OH)_2$, $PdO.H_2O$, or $Pd(OH)_2.H_2O$. Elsewhere, it was proposed that hydrous Pd oxides can be formed in acidic, basic, and neutral electrolytes; most of these conclusions are based only on the analysis of cyclic voltammetry profiles, and in some, they are also supported by other techniques [1].

References

1. Grdeń, M.; \Lukaszewski, M.; Jerkiewicz, G.; Czerwiński, A. Electrochemical Behaviour of Palladium Electrode: Oxidation, Electrodissolution and Ionic Adsorption. *Electrochimica Acta* **2008**, *53*, 7583–7598.
2. Rand, D.A.J.; Woods, R. The Nature of Adsorbed Oxygen on Rhodium, Palladium and Gold Electrodes. *J. Electroanal. Chem. Interfacial Electrochem.* **1971**, *31*, 29–38.
3. Lukaszewski, M.; Soszko, M.; Czerwiński, A. Electrochemical Methods of Real Surface Area Determination of Noble Metal Electrodes–an Overview. *Int J Electrochem Sci* **2016**, *11*, 4442–4469.
4. Pourbaix, M. Atlas of Electrochemical Equilibria in Aqueous Solution. *NACE* **1974**, *307*.
